# A Review of the Quality of Cardiometabolic Risk Monitoring Amongst Psychiatric Inpatients, and of Interventions to Reduce Their Long-Term Risk of Cardiovascular Disease

**DOI:** 10.1192/bjo.2022.433

**Published:** 2022-06-20

**Authors:** Thomas Cuthbert, Linh Ma

**Affiliations:** 1 Livewell Southwest, Plymouth, United Kingdom; 2 University Hospitals Plymouth NHS Trust, Plymouth, United Kingdom

## Abstract

**Aims:**

In Britain, individuals with severe mental illness die on average 15–20 years earlier than the general population. Their higher rates of cardiovascular disease contribute significantly to this. This audit reviewed how well cardiometabolic risk factors are screened for during inpatient admissions, and how frequently appropriate interventions are implemented for identified risk factors. It then assessed ways of improving current monitoring and interventions. We prioritised enhanced collaboration between patients and healthcare professionals, combined with formalising and systematising the physical health screening process.

**Methods:**

Bed coordination provided identification details of all patients admitted to an all-male acute psychiatric ward from 01/05/2019–31/08/2019. Each patient's record was reviewed to ascertain whether risk factors outlined in Lester UK Adaptation: Positive Cardiometabolic Health Resource were screened for. If a risk factor in this resource's “red zone” was identified, the patient's documentation was reviewed to see whether corrective action was attempted. Raw numbers and percentages of patients receiving any given physical health check were reviewed. For abnormal results, how many patients had appropriate action taken was then also checked.

**Results:**

63 patients were admitted, 50 of whom had a Rethink template completed. All physical health data (except blood results) were collected using the Rethink template.

41 patients smoked tobacco: seven accepted cessation support, 19 declined cessation support, and 15 were not offered support. 9 patients had no smoking status documented.

26 patients self-reported healthy lifestyles versus 24 who did not. Of these 24, 17 had no lifestyle intervention documented.

31 patients had a BMI > 25, of whom two were offered support, and 28 had no documented support.

12 patients were hypertensive, of whom three were offered further support, and eight had no further action documented.

44 patients were normoglycaemic, fifteen had no blood glucose test, and four had pre-diabetes/diabetes of whom one was offered further support.

32 patients had dyslipidaemia: one received further support, four were already on appropriate pharmacotherapy, and 27 had no further intervention documented. 25 had no bloods taken.

**Conclusion:**

Most patients had identifiable cardiometabolic risk factors: smoking, BMI > 25, poor lifestyle, dyslipidaemia, hypertension, hyperglycaemia (in decreasing order). Where risk factors were identified, intervention to address these risk factors and identification of barriers to supporting patients were lacking. COVID-19 may have changed the nature of admissions and health priorities. Structural changes were implemented, including changes to admission physical health assessments, introduction of well-man clinics, and improved communication between inpatient and community settings on discharge. A re-audit is pending.

